# Do Shy Preschoolers Interact Differently When Learning Language With a Social Robot? An Analysis of Interactional Behavior and Word Learning

**DOI:** 10.3389/frobt.2021.676123

**Published:** 2021-05-31

**Authors:** Nils F. Tolksdorf, Franziska E. Viertel, Katharina J. Rohlfing

**Affiliations:** Faculty of Arts and Humanities, Psycholinguistics, Paderborn University, Paderborn, Germany

**Keywords:** child–robot interaction, temperament, shyness, early childhood education, social robot, personality and behavior, word learning, individual differences

## Abstract

Temperamental traits can decisively influence how children enter into social interaction with their environment. Yet, in the field of child–robot interaction, little is known about how individual differences such as shyness impact on how children interact with social robots in educational settings. The present study systematically assessed the temperament of 28 preschool children aged 4–5 years in order to investigate the role of shyness within a dyadic child–robot interaction. Over the course of four consecutive sessions, we observed how shy compared to nonshy children interacted with a social robot during a word-learning educational setting and how shyness influenced children’s learning outcomes. Overall, results suggested that shy children not only interacted differently with a robot compared to nonshy children, but also changed their behavior over the course of the sessions. Critically, shy children interacted less expressively with the robot in general. With regard to children’s language learning outcomes, shy children scored lower on an initial posttest, but were able to close this gap on a later test, resulting in all children retrieving the learned words on a similar level. When intertest learning gain was considered, regression analyses even confirmed a positive predictive role of shyness on language learning gains. Findings are discussed with regard to the role of shyness in educational settings with social robots and the implications for future interaction design.

## Introduction

Recent years have seen a substantial growth in the applicability of social robots in educational learning environments with young learners. Examples are therapeutic settings (Boccanfuso et al., 2017; Cao et al., 2019), science learning (Causo et al., 2016; Park et al., 2017; Ioannou and Makridou, 2018), or language learning (van den Berghe et al., 2019; Vogt et al., 2019). More specifically, with the benefits of an embodied agent (Belpaeme et al., 2018), social robots offer versatile possibilities to engage children systematically in social interaction. Indeed, current research suggests that children accept social robots as trustworthy social actors from whom they can obtain reliable information (Breazeal et al., 2016; Vollmer et al., 2018; Oranç and Küntay, 2020). However, whereas these findings consider the demonstrated behavior displayed by children on average, individual differences in child–robot interactions have received little attention. Nonetheless, the way children enter into social interaction with their environment is mediated crucially by their individual temperament—specifically, by their shyness (Coplan and Evans, 2009). In fact, past research has demonstrated that a child’s shyness significantly influences her or his social behavior in familiar and unfamiliar contexts (Reddy, 2000; Evans, 2001; Coplan and Weeks, 2009; Crozier and Badawood, 2009; Feinberg et al., 2012; Colonnesi et al., 2014; Smith Watts et al., 2014). Additionally, shyness has a substantial impact on children’s performance in educational settings, insofar as shy children can either struggle to demonstrate their abilities in such situations or implicitly reduce their learning opportunities because they avoid social interaction (Evans, 2001; Spere et al., 2004; Smith Watts et al., 2014). Although some work has focused on how individual personality traits of adults affect interaction with a robot (Walters et al., 2005; Salter et al., 2006; Salam et al., 2017), research accounting for children’s behavior lags behind. Thus, our aim is to raise awareness in the area of child–robot interaction about how children’s personality traits such as shyness are reflected in their behavior when interacting with a social robot. Specifically, we aim to understand how the behavior of shy children might develop over a long-term interaction and influence learning gains with a social robot.

Extensive past research underlines the prevalence of shyness, indicating that up to 90% of the population experience shyness at some point in their lives with about 15% of individuals displaying a shyness that emerges in early development and remains stable across contexts (Zimbardo et al., 1975; Kagan, 1994; Schmidt et al., 2020). Shyness in children can be conceptualized as an increased and persistent behavioral inhibition in unfamiliar social situations or during perceived social evaluation that can result in withdrawal from interaction (Putnam et al., 2006; Rubin et al., 2009; Barker et al., 2014; see Schmidt and Buss, 2010, for a review). In developmental research, the effects of shyness are well documented, showing that the behavior of shy children toward their environment is reflected in both their verbal (Coplan and Weeks, 2009; Crozier and Badawood, 2009; Smith Watts et al., 2014) and nonverbal behavior (Reddy, 2000; Evans, 2001; Putnam et al., 2006; Feinberg et al., 2012; Colonnesi et al., 2014). For example, shy children are less talkative in familiar and unfamiliar contexts (Asendorpf and Meier, 1993; Evans, 1996; Crozier and Badawood, 2009) and display shyness through their facial expressions (e.g., by showing coy smiles) or their gaze behavior (e.g., duration of eye contact or gaze and head aversion; see Reddy, 2005; Colonnesi et al., 2014; Colonnesi et al., 2017). Importantly, recent research emphasizes that such expressions of shyness provide a positive and socially adaptive function for shy children within an interaction that enables them to regulate their emotions in unknown situations while also increasing prosociality and trust (Reddy, 2005; Colonnesi et al., 2013; Colonnesi et al., 2020).

Beyond the fact that shyness is reflected in children’s behavior toward an interaction partner, shyness also has an effect on the measured learning performance in educational contexts and particularly in the domain of language learning. Spere et al. (2004) and Spere and Evans (2009) have demonstrated that temperamentally shy preschoolers perform more poorly on both expressive and receptive vocabulary tests compared to nonshy children. Similarly, Hilton and Westermann (2017), recently investigated shy children’s retention abilities for learned word–object mappings and compared their learning outcomes with those of nonshy children. They looked at long-term word learning processes when the children were engaged in a word learning task: After a 5 min break, shy children did not retain any novel word they had formed during the learning situation; less shy children, in contrast, were able to retain the trained words. In line with work indicating that shy children are less likely to take risks in situations that are unknown or in which they are being evaluated (Addison and Schmidt, 1999; Levin and Hart, 2003), the authors suggested that shy children rely less on a guess in their responses, and that this might be detrimental in a situation in which they have to retrieve a novel word with ambiguous referents (Hilton and Westermann, 2017). Additionally, they argued that shyness may affect not only performance during an evaluation situation but also the immediate learning process—that is, due to shy children’s aversion to the unfamiliar, they might be less inclined to use novelty as a cue to the appropriate referent of a novel label when familiar/competitor objects are present. However, in this respect, it has been suggested that these differences in learning achievements could be context-dependent and not genuine, and that they should disappear under conditions that minimize anxiety or fear of evaluation (Spere et al., 2004; Hilton and Westermann, 2017). Along these lines, it should be borne in mind that conducting an experiment typically represents a fundamentally unfamiliar social situation for a child through, for instance, being exposed to unfamiliar people and unfamiliar settings. Thus, and against the background of evidence that shy children tend to show difficulties in experimental tasks (Crozier and Hostettler, 2003), it can be argued that the unfamiliar contextual environment during the testing situation might have influenced the recall abilities of shy children. Furthermore, Hilton and Westermann (2017), did not assess children’s general linguistic skills, although research has shown that existing linguistic knowledge should be considered when measuring word-learning processes (Stelmachowicz et al., 2004; McMurray et al., 2012). Therefore, investigations that include children’s linguistic abilities, are extended over a longer period of time, and are not limited to a single occasion could increase familiarity with the contextual environment and shed a more nuanced light on shy children’s learning outcomes in comparison to their nonshy peers. In sum, the currently available evidence shows that shy children’s behavior and learning is highly sensitive to contextual changes that may be particularly pronounced in social interactions such as in unfamiliar face-to-face encounters or during testing situations (Spere et al., 2004; Putnam et al., 2006; Matsuda et al., 2013).

Turning to the area of child–robot interaction, only a few attempts have been made to explore shy children’s behavior during interaction with a social robot, although possible implications of shyness for child–robot interaction in educational contexts are acknowledged (Baxter and Belpaeme, 2016; Baxter et al., 2017). Instead, most studies dealing with personality traits in this field examine approaches to provide the robotic system with the ability to automatically estimate the personality of a child based on predefined behavioral characteristics (Abe et al., 2014; Abe et al., 2017; Schodde et al., 2017; Abe et al., 2020; Sano et al., 2020) or to equip the robot itself with certain personality traits (Fischer et al., 2019; Calvo-Barajas et al., 2020). One of the few studies to address shyness in child–robot interaction investigated preschooler’s perceptions during a free-play situation in a kindergarten setting (Abe et al., 2014). In this study, parents evaluated their children’s shyness on a 5-point scale before estimating the social relationship between their child and the robot after a single “one-off” interaction. The study found that shyness clearly affected the relationship with the robot; and, according to the parents, one third of the shy children lacked a friendly relationship with the robot, whereas almost all nonshy children were friendly with the robot (Abe et al., 2014). Although examining parental reports about an experienced child–robot interaction is a proven methodological approach to assess and contextualize a child’s behavior (Tolksdorf and Rohlfing, 2020), how shy children actually behave within a child–robot interaction setting remains an open question. Additionally, given that familiarity with a situation strongly influences shyness (Rimm-Kaufman and Kagan, 2005), there is a general lack of a perspective that would include the long-term effects over multiple interactions.

Other studies that consider the effects of children’s individual personality traits are based on more implicit findings: Vogt et al. (2019) reported that in an educational child–robot interaction, certain preschoolers dropped out of the entire interaction due to shyness, or they needed additional support from the caregiver to successfully interact with the robot because they were reluctant or anxious even after an initial introduction to it. Shiomi et al. (2016) made a similar observation, showing that in a free-play situation, almost one quarter of the children hesitated to interact with the robot or avoided interaction entirely. The authors explained this rejection behavior as a result of the inhibition of these children (Shiomi et al., 2016). However, because they did not assess the children’s temperament, it is not clear whether their behavior can be linked to their shyness.

In a more recent work, Tolksdorf et al. (2020b) were the first to investigate which expressions of shyness are displayed by a child during an educational child–robot interaction. Their pilot study measured preschool children’s shyness with a standardized and validated questionnaire (Zentner, 2011) and analyzed children’s expressions of shyness toward the social robot across three sessions. In interaction with the robot, shy children not only behaved differently, but also changed their behavior over the course of the sessions: Although they showed significantly less positive behavior in the first interaction compared to the nonshy children, these differences disappeared in subsequent sessions. The authors argued that this could be explained in terms of increasing familiarity with the novel interaction partner, and that shy children might be able to overcome their reluctance to interact with a robotic system. In fact, these results are in line with work suggesting that young children react with uncertainty when facing a robot for the first time, and that they rely on an adequate introduction by a familiar caregiver to establish a beneficial learning environment (Rohlfing et al., 2020).

Taken together, these few studies indicate clearly that individual differences in the behavioral style toward a social robot exist in children, and they can be related to children’s shyness. However, any generalization across previous reports on the relation between shyness and children’s interaction behavior toward a robot is difficult because of the differences in the precise operationalization and assessment of shyness. Importantly, considering that social robots are being evaluated increasingly as learning partners, we do not know how far temperamental characteristics such as shyness might influence a child’s learning gain. Furthermore, although earlier studies evidenced that some behavioral effects in shy children’s behavior disappear when they are given the opportunity to familiarize themselves with the situation (Evans, 2001; Rimm-Kaufman and Kagan, 2005; Arbeau et al., 2010), the literature lacks a perspective focusing on how the behavior of shy children develops during a long-term child–robot interaction over multiple points in time and including multiple exposures to a test situation. Therefore, following up on our previous work, the current study aimed to address this research gap: We investigated the impact of shyness during an educational child–robot interaction by systematically examining children’s learning performance as well as their interaction behavior in terms of shyness markers and their signals of pleasure and distress toward the robot over the course of a long-term study on language learning.

In the present study, preschool-age children took part in a child–robot interaction over three consecutive learning situations followed by two test situations within a time period of two weeks. In line with recent accounts arguing that young children react with uncertainty when initially encountering a robot (Vogt et al., 2017; Rohlfing et al., 2020), we assumed that all children would interact with a certain reluctance at the beginning of the long-term interaction because they were faced with a novel and unfamiliar situation. Our main research interest was to explore how shy children’s behavior and language learning would develop with increasing familiarity during the sessions. We formulated the following hypotheses:

1. **(H1)** Based on the aforementioned work demonstrating shy children’s behavioral inhibition in unfamiliar social situations (Evans, 1996; Reddy, 2000; Putnam et al., 2006; Feinberg et al., 2012; Abe et al., 2014; Colonnesi et al., 2014; Vogt et al., 2019; Tolksdorf et al., 2020b), we expected that shy children would show less positive reactions (H1a) and more negative reactions (H1b) than nonshy children in both the learning situations and the test situations with the social robot just like they would be expected to do in human–human face-to-face interactions.
2. **(H2)** We also expected that negative reactions would decrease and positive reactions would increase with the repetition of a situation, especially among the shy children—both when there was a repetition of the learning situation (H2a) as well as when the test situation was repeated (H2b). This was because prior research has shown that the repetition of an interaction leads to an environment that becomes more predictable for a child while also increasing familiarity with the situation (Bruner, 1983; Rohlfing et al., 2016), and this might result in more positive expressions and minimize children’s social discomfort during the interaction (Colonnesi et al., 2014).
3. **(H3)** With regard to children’s language learning, we hypothesized that shy children would display lower learning achievements compared to nonshy children. This hypothesis is consistent with work indicating that shy children tend to perform on a lower level when their linguistic knowledge is tested in unfamiliar social situations (Spere et al., 2004; Hilton and Westermann, 2017). However, if growing familiarity with the testing environment allows shy children to feel more comfortable (Hilton et al., 2019), we expected that in the second test situation, the gap between shy and the nonshy children in displayed learning outcomes would decrease or disappear.

## Materials and Methods

### Participants

Thirty preschool children participated in the study. The data from two children were excluded because they did not participate in all sessions. This left 28 children (11 female, 17 male) aged 4.00–5.83 years (mean age = 4.98, *SD* = 0.48) for the final analysis. The children and their parents came from relatively high socioeconomic status backgrounds and were recruited from the wider Paderborn region (North Rhine-Westphalia, Germany). Recruitment was conducted in local kindergartens and libraries or through newspapers and our database of families willing to participate in our research studies. In addition, we assessed the level of parental education. None of the children or the parents had ever seen the robot before the experiment. Prior to their children’s participation, parents provided written consent and filled out a questionnaire on their child’s general and language development. Criteria for inclusion in the sample were: a) age between 4 and 6 years; b) normal general development such as normal sensory and cognitive skills; and 3) no developmental language delays. Additionally, detailed information regarding children’s language skills was obtained by measuring their receptive language abilities with a subtest of the standardized SETK 3–5 (Grimm et al., 2001) and their expressive vocabulary with the AWST-R (Kiese-Himmel, 2005). Parents were present during all interactions, but did not participate actively in the interaction. Children also provided verbal assent prior to taking part in the interaction, and the interaction could be discontinued at any time at no disadvantage to the child. Moreover, each child received stickers and a toy to thank them for their participation.

### Experimental Procedure

**FIGURE 1:**
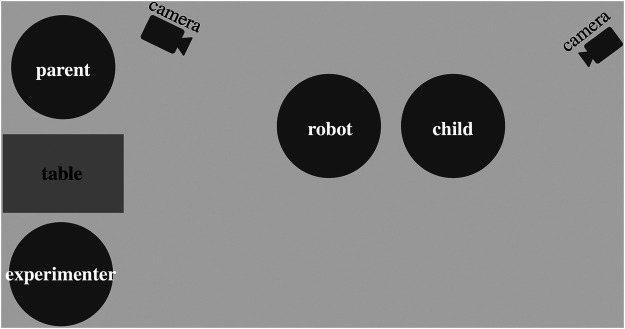
Setup of the study.

The children and their parents were invited to visit our laboratory at Paderborn University for four sessions within a period of two weeks. Each session lasted around 20–35 min, and all sessions were recorded on video. Each participating child was accompanied by one parent. Figure 1 displays the seating arrangement. The experimenter operated the robot, avoiding any interaction with either parent or child. In addition, parents were instructed to avoid talking to their child during the experimental part of the child’s interaction with the social robot.

Based on previous work and ethical considerations (Vogt et al., 2019; Tolksdorf et al., 2020a; Tolksdorf and Mertens, 2020), we conducted a warm-up phase with each child and her or his parent before the learning situation with the robot. This introduced the robot to the children in a comfortable way with their caregiver as an available resource (Manner et al., 2018; Tolksdorf et al., 2020a) and reduced the novelty effect (Kanero et al., 2018). During the warm-up, the experimenter first introduced the robot in a powered-off state to the child and parent and explained its functions. For example, it was explained that the robot can talk and move with the help of small motors, because a pilot study had shown that some children were surprised when they heard that the robot’s movements were loud. In a second step, children and parents were further familiarized with the capabilities of the robot: The robot introduced itself and performed a short game by imitating animal movements and asking the child, the parent, and the experimenter to repeat the movements. Although the experimenter structured the situation and was the main interaction partner for the child, parental involvement was considered as an important element during the warm-up phase, because prior work has demonstrated that young children may rely on the emotions with which their familiar caregiver interprets the ongoing situation, especially during first encounters with a social robot (Rohlfing et al., 2020; Tolksdorf et al., 2021). After the game was completed, the robot said goodbye for the moment and announced that it had prepared a story that it wanted to share with the child. Subsequently, the experiment started and the script for the first learning situation was launched.

**FIGURE 2:**
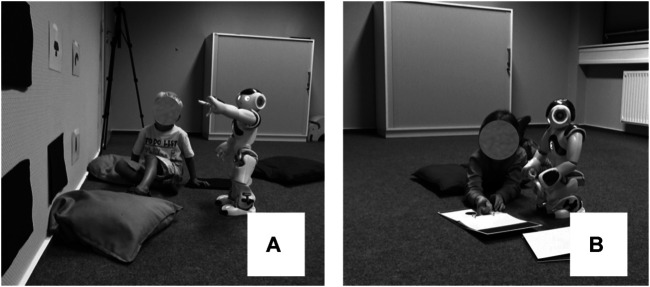
The learning situation **(A)** and test situation **(B)**.

When designing the learning situation, we were guided by theoretical concepts of learning postulating that interaction partners jointly co-construct the communicative situation in a goal-oriented way (Rohlfing et al., 2016; Rohlfing et al., 2019). Therefore, we chose a setting that included activities with which preschoolers are familiar. Specifically, the robot told the child a story that had been created to frame the learning situation. The story contained the plot of the robot’s trip to Paderborn University and the things it had seen on its journey. This narrative served as the context in which the children encountered six novel words (color adjectives) during the interaction. The referents of the novel words were presented as pictures hanging on the wall. They were covered by a small cloth, and the robot asked the child to uncover them one by one over the course of the interaction (see Figure 2A). This context was also chosen because past work has shown that the context of a story is particularly conducive to children’s learning of new words (Horst, 2013; Nachtigäller et al., 2013).

Furthermore, in order to render the robot’s interaction behavior child-oriented and to fulfill the important role of multimodal joint activities (Rohlfing et al., 2019; Tolksdorf and Mertens, 2020), the robot also performed a number of actions such as accompanying the novel words with pointing gestures to coordinate the child’s attention and establish a shared reference. In the same way, the robot coordinated its gaze between the child and the referents of the target words. Additionally, after naming the first four target words, the robot also walked with the child to the two remaining target referents in order to make the situation more natural and to take advantage of the physical presence of the robot (van den Berghe et al., 2019). Once the robot had finished the story, it thanked the child and said goodbye.

Subsequently, in the second and third sessions, a similar learning situation with the robot took place again in which the robot shared the story, and the children were exposed to the novel words.

Following the third learning situation in the third session and a 5 min break, children’s learning achievements were assessed by testing their ability to generalize the acquired knowledge. In this generalization task, comprehension was defined as the child’s ability to extend or transfer the target words to new objects, and whether they were able to transfer the learned pattern of word formation to new colors when presented with separated units of a compound. This method ensured assessment of whether the children were able to transfer their knowledge to other objects and whether their knowledge was stable. We used a routinized activity for children and embedded the test procedure within a shared picture book reading situation (Grimminger and Rohlfing, 2017). In this test, the child was asked to turn the pages while the robot talked about the pictures with the child and elicited the trained words (see Figure 2B).

Two to three days later, the last, fourth session was conducted, in which a delayed test using the same procedure was administered to assess the children’s knowledge again.

### Stimuli

Our study used the Nao robot from Softbank Robotics. This is a small, toy-like, humanoid robot used widely in child–robot interaction studies (Belpaeme et al., 2018). It is 58 cm high with 25 degrees of freedom. Teleoperation was employed to enable the robot to act contingently (Kennedy et al., 2017). We implemented the behaviors in the NAO robot by using *Choregraphe* and used the integrated text-to-speech production of the robot with German language enabled and speech reduced to 85% speed to achieve a more natural pronunciation. The target words imparted by the robot were spoken at a speed of 75% in order to emphasize them verbally. The target items consisted of six morphologically complex words (noun–adjective compounds such as “quince yellow [*quittengelb*]”) that represented different colors as features of different objects. Each item was presented on a picture measuring 14.8 × 21.0 cm.

### Coding of Children’s Behavior

**FIGURE 3:**
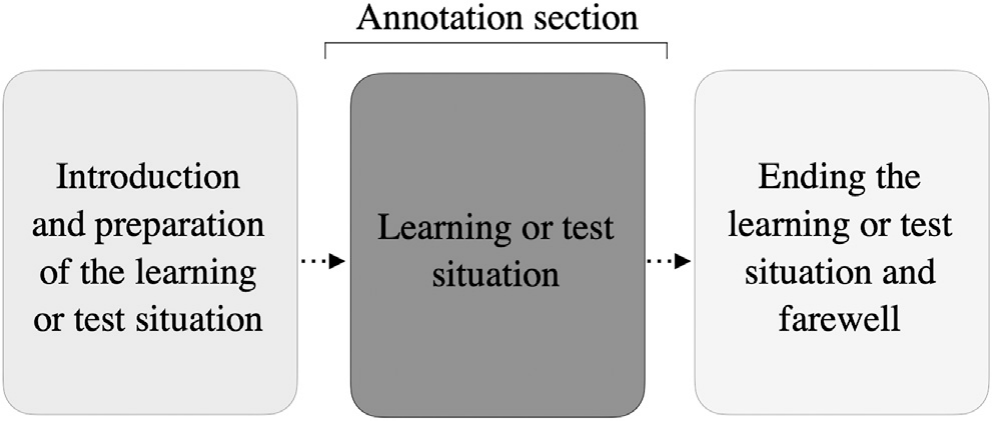
Sequence of the interaction during sessions and annotated section.

Because we were interested in children’s behavior during all the learning and testing situations with the social robot, we followed Colonnesi et al. (2013) and Colonnesi et al. (2014) and coded positive as well as negative reactions with and without signs of aversion such as body or head aversion. Positive behaviors of the children were measured by, for example, smiling identified by raising the corners of the lips, constriction of the eyes, raising of the cheeks, or opening of the mouth. Negative behaviors were frowning or sad facial expressions. When these behaviors were accompanied by an aversion (of gaze, head, and body), they fell into the category of positive or negative expressions of shyness. We measured this behavior across the time period of the interaction during which the robot a) shared the story and taught the new words (learning situation T 1–3) and 2) tested the child within the shared picture book situation (Test 1 and Test 2), (see Figure 3).

We decided to analyze this sequence because, at this stage, all children had already achieved a certain familiarity with the new interaction partner, and our focus was on investigating development across different sessions. Additionally, the analyzed sequence represented the main part of the interaction, whereas a welcome or farewell situation would reflect a different social situation with its own contextually appropriate social behaviors (Vaughn and La Greca, 1992). Examining this sequence is particularly relevant, because it provides an opportunity to understand how shy children interact during a learning situation with a social robot. Because the duration of the interactions varied slightly between children, children’s behavior was expressed in proportion per minute. To evaluate coding reliability, two coders independently coded a random subset of 15% of the data. We used Cohen’s Kappa to measure the agreement between the coders for positive and negative reactions, expressions of shyness, and children’s aversion. The mean Kappa values were between 0.88 and 0.94, indicating a high level of internal consistency.

### Assessment of Naming Performance

Following recent methodological accounts (Rohlfing and Grimminger, 2019), we chose to assess children’s word-learning performance in detail on different linguistic layers rather than in a binary way (e.g., only correct or incorrect). To provide a measure of word learning, we created a composite score by averaging each of the children’s naming performances in percentages on a phonological, morphosyntactic, and pragmatic–semantic level. Thus, on a phonological level, we calculated the proportion of correctly produced syllables of a target word. On a morphosyntactic level, and independent of the semantic meaning, we assessed the sophistication with which the children constructed a noun–adjective compound and distinguished between no compounding, partial compounding, and fully correct compounding. Finally, we evaluated each child’s response on a pragmatic–semantic level, differentiating between no response, a semantically adequate response, a partial retrieval of the target word, and a fully correct retrieval. The maximum composite score that could be achieved in each test session was 3, which would reflect a fully accurate performance on each of the three linguistic levels.

### Assessment of Shyness and Shyness Questionnaire

To assess the children’s degree of shyness, we used the Inventory on Integrative Assessment of Child Temperament (German: *IKT*—*Inventar zur integrativen Erfassung des Kind-Temperaments*, Zentner, 2011). This is a standardized questionnaire that is widely used in clinical practice and is specifically designed for the age group addressed. The IKT has been validated with a normative sample of over 4,400 children, possesses convergent validity with equivalent English-language temperament diagnostics (e.g., the CBQ by Rothbart et al., 2001), and measures the temperament of 2- to 8-year-olds on five levels based on the integrative approach of Zentner and Bates (2008). With their approach, the authors pursue the goal of overcoming the manifold conceptions of child temperament research and providing a questionnaire that is valid across theories (Zentner and Bates, 2008). The levels comprise shyness (behavioral inhibition), susceptibility to frustration, activity level, attention span/task persistence and perceptual sensitivity. In our study, caregivers were asked during the first session (T1) to fill out the questionnaire and to estimate how often their child shows a described behavior using a 6-point Likert scale ranging from 1 (*never*) to 6 (*always*). This included behavioral aspects such as “hides behind her mother when she meets strangers.” Based on the raw scores obtained from the responses to the questions, the evaluation procedure of the test requires a conversion into percentile ranks to allow an adequate interpretation of the child’s temperament according to age and gender in relation to the normative sample of the test. The higher the percentile rank value, the shyer the child, with the minimum and maximum value being 0 and 100 respectively. In this vein, the IKT allows children to be considered as notably shy if they have a clearly above-average score of over 75. Additionally, the shyness scale used in the test procedure provides a good internal consistency (*α* = 0.81). In the normative sample, the agreement of both parents as a measure of interrater reliability was clearly above average (*r* = 0.73) and thus highest on the shyness level compared to the other levels (Zentner, 2011). Finally, in accordance with the evaluation procedure and based on the percentile ranks obtained, we grouped our sample into two levels: nonshy (*n* = 18) and shy (*n* = 10).

## Results

Table 1 presents an overview of all demographic data as well as the group means and standard deviations of the language and temperament measures.

**TABLE 1:** Mean participant characteristics for shy and nonshy children and standard deviation (SD).

Independent variable	Total (*N* = 28)	
	Nonshy (*n* = 18)	Shy (*n* = 10)
Age in years	*5.0 (0.5)*	*5.0 (0.4)*
Parental education level^1^	4.7 (*1.0*)	*4.1 (1.3)*
Gender		
Female	7 (39%)	4 (40%)
Male	11 (61%)	6 (60%)
SETK 3–5 sentence comprehension^2^	53.9 (*8.2*)	46.3 (*8.3*)
AWST-R expressive vocabulary	60.1 (*11.8*)	51.9 (*10.6*)
IKT shyness score^2^	40.4 (*24*)	87.2 (*6.3*)

1 Level of parental education on a scale from 1 (*lowest*) to 6 (*highest*).

2 Converted raw values into percentile ranks.

### Positive and Negative Shyness Reactions

A statistical analysis of shyness markers was not possible, because values tended toward zero in almost all training and testing periods. Therefore, we focused on analyzing the behavioral markers indicating pleasure and distress described in the following. In the *Limitations* section, we shall discuss some issues that may have led to the very rare occurrence of typical shyness reactions.

### Expression of Pleasure

First, we wanted to know how far positive reactions were more likely in the nonshy group. We also assumed that due to the familiarity of the situation, positive reactions would increase over time in both groups, but especially in the shy group that would start off being more reserved but become more uninhibited over the course of the sessions.

Parametric statistical tests could not be used to analyze this dependent variable due to nonnormally distributed data and the small sample size. Therefore, we used the ANOVA type statistic (ATS)—a nonparametric equivalent of a mixed ANOVA (Akritas et al., 1997)—performed with the software R (package: nparLD, Noguchi et al., 2012). The ATS is regarded as a distribution-free test, but is mathematically more appropriate than classical rank-sum statistics such as Wilcoxon’s rank-sum test. The test statistic is quite similar to ANOVA’s *F* tests and exactly meets the *α* level while being conservative. It has been applied in developmental studies (Viertel, 2019; Tolksdorf et al., 2021). In addition, the ATS can tolerate unequal group sizes in the sample and is robust when studying longitudinal data because it considers their progression over time rather than comparisons between groups at each timepoint that may inflate type I error. The relative treatment effect (RTE) is a measure of the effect size and is estimated based on the actual sample. It can be determined for main effects as well as for interaction effects—that is, even for multifactorial designs with repeated measurements (Noguchi et al., 2012) as in the present study. The value of the relative effect RTE ranges between 0 and 1, whereby the occurrence of 0 and 1 means completely different conditions (e.g., for the shy and nonshy group); 0.5 indicates that the conditions do not differ at all (Brunner and Munzel, 2002; Noguchi et al., 2012).

A significant main effect demonstrated that nonshy children (*Mdn* = 1.14, *IQR* = 1.67, *RTE* = 0.57) used positive behaviors significantly more often than their shy peers (*Mdn* = 0.50, *IQR* = 1.37, *RTE* = 0.37), *F*(1.00, 17.33) = 6.51, *p* < 0.05, regardless of the situation (learning and testing). This supported Hypothesis H1a.

Additionally, the ATS revealed a highly significant main effect of time, *F*(3.00, ∞) = 7.77, *p* < 0.001. Positive reactions were highest at the beginning of the training of novel words (T1: *Mdn* = 1.71, *IQR* = 1.54, *RTE* = 0.61), decreased steadily over the course of training (T2: *Mdn* = 1.27, *IQR* = 1.06, *RTE* = 0.56; T3: *Mdn* = 0.63, *IQR* = 0.97, *RTE* = 0.40), and then remained relatively stable in both tests (Test 1: *Mdn* = 0.55, *IQR* = 1.15, *RTE* = 0.39; Test 2: *Mdn* = 0.60, *IQR* = 1.03, *RTE* = 0.40). Post hoc tests were applied with Bonferroni corrections. A significant decrease of positive reactions was detected from training T1 to Test 1 (difference = 34.0) and Test 2 (difference = 38.0) respectively, *χ* ^2^(4) = 15.86, *p* < 0.01. In all cases, the critical difference was 33.21. This observed development of positive reactions contradicted Hypotheses H2a and H2b that positive reactions would increase with the repetition of a learning or test situation.

Moreover, contrary to our hypotheses, there was no interaction between time and shyness level, *F*(3.00, ∞) = 0.65, *p* = 0.58. Hence, shy children remained reserved over time by demonstrating fewer positive reactions such as smiles over the course of both the learning and the testing situations.

### Expression of Distress

In a further step, we asked how far the frequency of negative reactions differed between shyness groups and training conditions depending on the time of training and testing situation. We hypothesized that the shy group would show negative reactions more frequently (H1b), but that these would decline more rapidly among the shy group than among the nonshy group in both the training situation (H2a) and the testing situation (H2b). Again, we refrained from using parametric statistics and used the ATS.

The ATS revealed a highly significant effect of time, *F*(3.34, ∞) = 14.22, *p* < 0.001. It was evident that negative reactions were stable across the first two training sessions (T1: *Mdn* = 0.45, *IQR* = 1.04, *RTE* = 0.58; T2: *Mdn* = 0.51, *IQR* = 0.75, *RTE* = 0.56), decreased during last training (T3: *Mdn* = 0.00, *IQR* = 0.32, *RTE* = 0.29), briefly increased during the first test (Test 1: *Mdn* = 0.57, *IQR* = 1.00, *RTE* = 0.62), and finally, flattened in the second test (Test 2: *Mdn* = 0.19, *IQR* = 0.20, *RTE* = 0.35).

There was also a trend toward a significant difference between shyness groups, with shy children (*Mdn* = 0.16, *IQR* = 0.50, *RTE* = 0.42) demonstrating negative reactions less frequently than nonshy children (*Mdn* = 0.33, *IQR* = 0.64, *RTE* = 0.55) in all training and test situations, *F*(1.00, 18.21) = 3.33, *p* = 0.07. Therefore, we rejected Hypothesis H1b.

However, both factors need to be interpreted in relation to each other, because there was a significant interaction effect between time and shyness group, *F*(3.34, ∞) = 2.58, *p* < 0.05. Post hoc tests showed that shy children expressed negative reactions less frequently, especially during the second training (*Mdn* = 0.24, *IQR* = 0.46, *RTE* = 0.41) as well as during the second test (*Mdn* = 0.12, *IQR* = 0.17, *RTE* = 0.26) compared to nonshy children (T2: *Mdn* = 0.66, *IQR* = 1.43, *RTE* = 0.71; Test 2: *Mdn* = 0.26, *IQR* = 0.19, *RTE* = 0.45). Differences during training (*W* = 144.5, *p* < 0.01, *r* = −0.49) and testing (*W* = 146.5, *p* < 0.01, *r* = −0.51) were both very significant. Thus, the result that only shy children showed negative reactions significantly less often when a situation was repeated supported Hypotheses H2a and H2b.

Additionally, in the shy group, multiple comparisons across time revealed that negative reactions decreased significantly from first to third training (T1 *vs.* T3: *χ* ^2^(4) = 16.17, *p* < 0.01), with the observed difference of 20.5 exceeding the critical difference of 19.85. In the nonshy group, a significant reduction of negative reactions from T2 to T3 could be identified in the training situations, *χ* ^2^(4) = 19.34, *p* < 0.001. The observed difference was 30.5 and thus higher than the critical difference of 26.63. Surprisingly, in the group of nonshy children, there was a significant increase in negative reactions from the last training session (T3) to the first testing session (Test 1) with an observed difference of 29.5. In the shy group, a tendency toward an increase was identified based on the observed difference of 19.5 (critical difference: 19.85). In conclusion, over the course of the training phase for novel words (T1–T3), negative reactions decreased in both groups, but this familiarization effect occurred more rapidly in the group of shy children.

Summarizing the frequency of both positive and negative reactions in the learning and test situations, it can be concluded that the group of shy children was generally less expressive compared to the group of less shy peers.

### Shyness Score and Condition as Predictors of Word Learning

Finally, we focused on shy children’s word learning, which we assumed would be less successful than that of nonshy children––but only during the first test session (H3). Children’s word learning was measured based on the calculated linguistic composite score (0–3) reflecting their performance during the test tasks (cf. section *Assessment of Naming Performance*). As hypothesized, a strong trend could be observed during the first test, and shy children (*Mdn* = 0.51, *IQR* = 0.36, *range* = 0–1.52) tended to be less successful in retrieving the taught words than their nonshy peers (*Mdn* = 0.64, *IQR* = 0.27, *range* = 0–2.54), *W* = 122, *p* = 0.06, *r* = −0.35. Furthermore, also as hypothesized, both groups (shy: *Mdn* = 0.48, *IQR* = 0.27, *range* = 0–2.54; nonshy: *Mdn* = 0.72, *IQR* = 0.72, *range* = 0–2.06) did not differ significantly in their success at retrieving the words during the second test session (*W* = 119.5, *p* = 0.16, *r* = −0.26), which was a repetition of the first test a few days later.

Last of all, we wanted to go beyond the dichotomous group comparison and ask how the entire range of the shyness spectrum as an influencing factor predicted a gain in word learning when the children were already familiarized with the experienced testing situation during Test 1. As described, the testing situation took place in two different sessions (Test 1 and Test 2). Thus, we calculated the gain in word learning by measuring the difference scores between the first and the second test as a metric of learning gain. As a predictor variable, we did not use the categories *nonshy* and *shy*, but the calculated percentile ranks taken from the IKT described above (cf. section *Assessment of Shyness and Shyness Questionnaire*) that represented the full shyness spectrum and would allow us to take a closer look at the link between temperamental characteristics and word-learning processes.

Therefore, our hierarchical regression model included the shyness score as a predictor of a gain in word learning (first step). Moreover, when measuring children’s word learning, it is known from the literature that existing linguistic knowledge should be taken into account because it contributes to word learning success (Stelmachowicz et al., 2004; McMurray et al., 2012). Therefore, in the second step, we integrated a language measure of children’s receptive linguistic abilities (SETK 3–5 subtest sentence comprehension, cf. Table 1).

**FIGURE 4:**
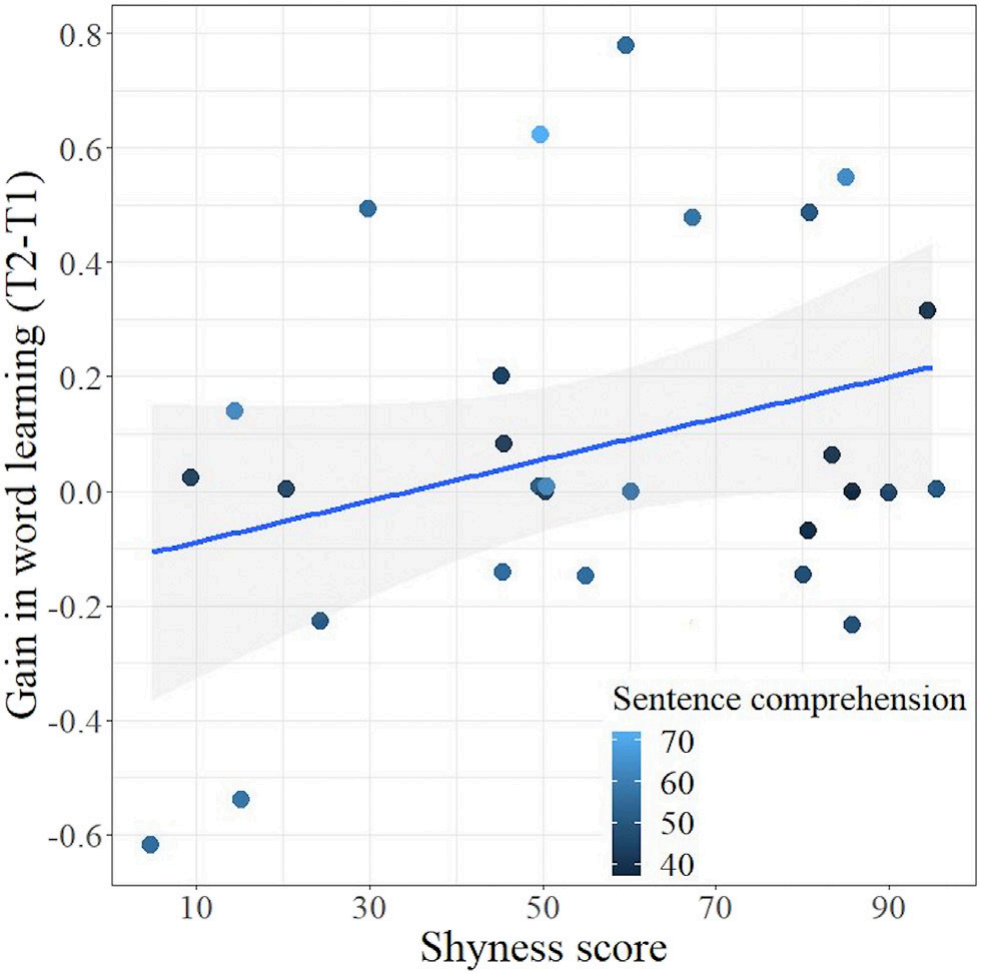
Scatterplot with linear regression line (including 95% confidence interval) illustrating the predictive relation between level of shyness and gain in word learning (difference scores of word learning between T2 and T1). Receptive linguistic skills are integrated as converted percentile ranks.

In Step 1, the model did not differ significantly from zero, *F*(1, 26) = 2.72, *p* = 0.11, with shyness level accounting for 9.48% of the variance in learning gain. In Step 2, the model approached statistical significance and accounted for an increased portion of gain in word learning, *F*(2, 25) = 3.18, *p* = 0.06, and could explain 20.28% of the variance. Shyness scores related significantly to a gain in word learning (*B* = 0.005, *t* = 2.18, *p* = 0.04) and uniquely contributed 14.63% to the total variance of our dependent variable (see Figure 4). The shyer the children were, the higher their growth of learning as suggested by the significant positive semipartial correlation (*r* = 0.38, *p* = 0.05). In terms of receptive linguistic abilities, we detected a nonsignificant trend (*B* = 0.01, *t* = 1.84, *p* = 0.08) toward a positive association between children’s receptive language and word learning gain (*r* = 0.33, *p* = 0.09), whereas language abilities independently accounted for 10.96% of the variance. A comparison of both models confirmed that in Step 2, *R* ^2^ tended to change, *F*(1, 25) = 3.38, *p* = 0.08.

Finally, we checked whether other factors, which had not been considered in the multiple regression model before, were associated with word learning. Neither age (*r* = 0.12, *p* = 0.27, one-sided) nor expressive vocabulary collected by the AWST-R (*r =* −0.02, *p* = 0.55, one-sided) related significantly to a gain in word learning. In summary, this means that shy children showed greater gains in word learning than children who were less to moderately shy, but only when their receptive skills were considered in the model as well.

## Discussion

The present study examined how shy children, compared to nonshy children, enter into and maintain social interaction with a social robot during a word-learning educational setting, and how children’s learning outcomes relate to their temperament. The study was motivated by previous research suggesting that shy children exhibit marked differences in their learning and social behavior toward interaction partners including social robots. In this respect, the contribution of our study is twofold: First, despite the importance of shy children’s familiarization with a situation (Evans, 2001; Rimm-Kaufman and Kagan, 2005; Arbeau et al., 2010), we could not find any research investigating the behavior of shy children over a long-term interaction with a social robot. Therefore, we systematically assessed children’s personality trait of shyness and investigated their behavior. More specifically, we specified markers of shyness and their signals of pleasure and distress toward the robot during multiple learning situations and during repeat testing situations in which children’s learning was evaluated. Second, this study expands previous research by identifying different learning trajectories linked to the effects of shyness on children’s learning performance. Whereas some work in human–human interaction indicates that shy children tend to perform more poorly in unfamiliar test procedures (Spere et al., 2004; Hilton and Westermann, 2017), findings on the impact of shyness on learning outcomes in the field of child–robot interaction are scarce.

Overall, results show that shy children not only interact differently with a robot compared to nonshy children, but also change their behavior over the course of the sessions. In fact, shy children interacted significantly less expressively with the robot in general. With regard to children’s learning outcomes, shy children tended to score significantly lower on the first test, although they were able to close this gap during the second test, resulting in all children retrieving the learned words on a similar level. Surprisingly, we could even observe that once a certain familiarization with the test procedure was established, shyness related significantly to a gain in word learning when the receptive linguistic abilities were taken into account at the same time. In the following subsections, we shall interpret our findings one at a time.

### Children’s Expressions of Pleasure and Distress Toward the Robot

As expected, and in accordance with previous literature (Reddy, 2000; Putnam et al., 2006; Feinberg et al., 2012; Abe et al., 2014; Vogt et al., 2019; Tolksdorf et al., 2020b), we found that shy children were more reserved in their positive reactions toward the robot compared to their nonshy peers in all learning and testing situations. This lower level of positive reactions could be explained by the typical expressive pattern of shyness in novel social situations (Reddy, 2000; Colonnesi et al., 2014)—that is, shy children tend to display reduced emotional reactions and be more inhibited in unfamiliar social interactions (Poole and Schmidt, 2019). Surprisingly, we did not find support for our hypothesis that the number of positive reactions would increase with the repetition of a situation and increasing familiarity. Instead, our results revealed an opposite trend. Overall, the frequency of occurrences of positive reactions was most pronounced in the first two learning situations and then decreased steadily, reaching its lowest level in the final two test situations. With regard to the nonshy children, the results might be explained by the fact that precisely the novelty of the situation (e.g., the novel interaction partner and storytelling setting as suggested in Kanero et al., 2018) led to higher levels of engagement that were reflected in more positive reactions. In this vein, our results corroborate existing research in the area of child–robot interaction demonstrating that with the increasing duration of an interaction and repetitive behavior of a robot, children’s engagement in terms of enjoyment could drop (de Wit et al., 2020). Thus, the repeated sessions with the robot might have led to a habituation effect and resulted in decreasing positive reactions. However, we could not observe a significant change in the expressiveness of the shy children’s positive reactions over the course of the sessions, suggesting that their display of enjoyment of the situation remained constant on a low level, even during the learning situations.

Regarding the negative reactions as an expression of discomfort in a new situation, results show that behaviors such as frowning or narrowing the eyes reduced significantly more quickly over time in the group of shy children, probably due to the fact that they had become accustomed to the learning and testing situation. In particular, when the shy children were already familiarized with a specific setting (first learning situation or first test), reactions of distress were significantly lower in comparison to nonshy children. A decrease in reactions of distress also occurred in the nonshy children–but at a slower rate. In addition, in the latter group, the significant increase in negative reactions stood out when the setting (from learning to testing) and the associated demands on the children changed.

This finding has strong implications not only for studies evaluating social robots as interaction partners in educational settings, but also for studies examining word learning processes or other cognitive abilities in young children in general. Negative reactions (of distress) relate positively to general and social anxiety (Colonnesi et al., 2014), and negative shy reactions are associated with physiological changes that can activate the fight-or-flight system (Colonnesi et al., 2020) that consequently inhibit adaptive behavior and cognitive processes. During learning with others, they are often inhibited, which is reflected in deviant attentional processes (Hilton et al., 2019) as well as infrequent eye gaze to the other (Putnam et al., 2006). Thus, in our view, a warm-up that usually takes place a few minutes before the training or testing may not be sufficient for a specific population such as very shy children. Considering the fact that especially nonshy children expressed the highest proportion of reactions of distress during the first two learning sessions (T1 and T2), our conclusion is not exclusively of relevance for shy children, but also for children with different temperament traits.

### Children’s Word Learning

Our third hypothesis addressed how word learning in shy children differs from that in their nonshy peers at different time points. Motivated by the literature (Hilton and Westermann, 2017), we assumed that shy children would be less successful at retrieving the learned words than nonshy children, especially in the first test situation. We further hypothesized that the difference between groups would decline during the second test due to familiarization with the test procedure. At first impression, the performance of both shy and nonshy children in the word learning tests seems to be in line with our hypothesis, because in both tests, shy children were less successful in retrieving the trained words. Although the difference was marginally significant in the first test session, it disappeared in the second session. However, we considered that a further, more nuanced approach was needed, because there could be legitimate objections to our categorization into shy and nonshy. In particular, because children who were not at all shy fell into the same category as children who were on the threshold of being very shy (according to the questionnaire), we regarded our dichotomization as not being precise enough. Therefore, we conducted a regression analysis to determine the relation between the degree of shyness and word learning. Moreover, our multilevel model also took into account the children’s linguistic knowledge, and we were able to show that, in addition to a shy temperament, the children’s receptive abilities also tend to contribute to a success in word learning. Surprisingly, past studies on learning words with shy children (Hilton and Westermann, 2017; Hilton et al., 2019) or language learning studies with robots (Vogt et al., 2019) have not considered these linguistic abilities; therefore, our study marks another novelty in this field.

Interestingly, the children who were the shyest according to their parents made the largest gains in word learning, whereas those with the lowest shyness scores remained stable or even scored lower on the second test. This contrasts with results obtained in studies concluding that shy children are a) less likely to learn and retain new words (Hilton and Westermann, 2017) or b) have poorer productive vocabularies (e.g., Crozier and Hostettler, 2003). Additionally, these prior studies drew their findings from a single test—that is, word learning or vocabulary of shy children is typically assessed in a new environment with unknown people without any prior familiarization with the situation. This raises the question whether the shy children’s test performance would be similar to that of nonshy children if they were already familiar with the situation (as realized in our study). As described above, shy children are afraid of being evaluated in unfamiliar situations, which consequently inhibits their performance, as evidenced by studies that a) examine vocabulary less invasively, for example by parents or in familiar school settings (Spere and Evans, 2009); or 2) in more anonymous group settings (Crozier and Hostettler, 2003); but also by studies that 3) determine the receptive vocabulary (Evans, 1996). These studies conclude that the linguistic performance of shy children does not differ from that of nonshy children. Therefore, based on our data, we assume that the shy children were more confident during the second testing and verbally expressed themselves more often once they were familiarized with the exact procedure and the demands of the test situation.

As well as the repetition of the testing situation, another possible reason could contribute to a short familiarization: the pragmatic frame of the situation of joint book reading that is structurally anchored in the test situation (Rohlfing et al., 2016). Accordingly, we can assume that the test situation recedes into the background or is not perceived as such by the children, so that their cognitive abilities can unfold (Rohlfing et al., 2016). Additionally, because our robot was introduced as a coequal peer (Kory Westlund et al., 2016; Vogt et al., 2017), it might not be perceived as an authoritative character, as other examiners are often perceived to be by shy children, but rather as an interaction partner who elicits learned words or, in general, verbal responses in a familiar situation. Therefore, shy children may feel less evaluated during an interaction with a robot, especially in terms of their performance, and this could lead them to be less cognitively inhibited and more confident when attempting to guess an answer.

## Limitations

Finally, it is worth discussing why shyness markers appeared so rarely during training and testing that we were unable to carry out any analysis on this basis, but instead concentrated on behaviors expressing the children’s emotionality of pleasure and distress but without any aversion. In 4-year-olds, state shyness is measured differently than in our study: Children are asked to sing a song on a stage in an unfamiliar location in front of a small group of strangers and their caregivers––the so-called performance task (Colonnesi et al., 2017). This scenario differs clearly from our setting in which the child is neither required to perform nor is her performance exposed to the other’s attention. Hence, one objection might therefore be that the setting applied in our study failed to elicit shy reactions (Colonnesi et al., 2014). On the other hand, many factors triggering shyness reactions existed in our setting such as unknown interaction partners (and among them a robot), a new situation with novel words in an unfamiliar location, and the recall of a previously taught object of learning. Interestingly, the age of the children, which ranged from 48 to 70 months, may have contributed to the fact that (in particular negative) shyness reactions were rare–even in shy children. In this context, Colonnesi et al. (2020, p. 49) discuss their results with 72-month-olds: “A possible explanation is that later individual socio-cognitive development (e.g., advanced social cognition, social skills) and effortful control are responsible for less frequent and more regulated shy reactions.” Instead, preschoolers express their shyness by reactions of distress and avoidance as well as ambivalence in their behavior–a pattern we were able to identify in our study as well. In this respect, Colonnesi et al. (2014) demonstrated that positive and negative reactions correlated positively with the corresponding shyness markers, thus concluding that they represent expressions of the same emotion but with a different emotional valence. In this context, it should be emphasized that we used a parental assessment of trait shyness based on a questionnaire. We regarded this as being the most reliable and valid for our study design rather than concentrating merely on state shyness measured in a specific situation. Finally, it is also important to recognize that one limitation of this study is its relatively small sample size. However, we wish to highlight that according to recent methodological findings, conducting long-term studies with small samples over multiple sessions while repeatedly measuring the variable of interest over time enhances replicability and robustness (Smith and Little, 2018). In this vein, the approach adopted here allows a particularly nuanced view of children’s behavioral development. Additionally, the statistical procedures used were rather conservative in terms of determining significant effects and tolerant of both small sample sizes and unequal groups (Noguchi et al., 2012). Lastly, although the sample size used is consistent with previous studies utilizing a similar paradigm (McGregor et al., 2009) and we found clear differences between groups, more research is needed to further validate our findings.

## Conclusions and Future Considerations

In conclusion, the results presented here offer new input for not only research with social robots in educational settings and the design of future learner–robot interactions but also for evaluating and measuring children’s achieved learning outcomes. In fact, most past research in child–robot interaction has tested hypotheses by comparing average effects across the sample but ignored that effects may vary across individuals depending on existing intrinsic factors such as shyness. In this vein, the present study provides evidence that shy children do indeed demonstrate a distinctive behavior in terms of their interactions with a robot as well as their language learning. Our findings show that it is important to include the learner’s temperamental characteristics, such as shyness, during child–robot interactions to inform the use of robots in the educational field. In this regard, current research strongly suggests the need to consider children’s temperament in everyday practice in institutional settings such as kindergarten or school and provide a supportive climate for a variety of children and temperament types (Ann Sealy et al., 2021). Whereas approaches to automatically assessing a child’s personality based on predefined behavioral traits have made substantial progress (Abe et al., 2017; Abe et al., 2020; Sano et al., 2020), the present study has taken first steps to determine which behaviors can actually be observed in shy children and how they develop in the long term over a period of several sessions.

From our results, we can derive some crucial aspects for designing future interactions with child-oriented social robots: On the one hand, testing shy children requires a greater familiarity with the situation, and this can be achieved by a prior acquaintance with the interaction partner, the location, and the items. For example, an extended warm-up session in advance of a learning *and* testing situation could be a solution in future interaction designs. This could be conducted individually in addition to carrying out an introduction in a group of children. Additionally, based on our data, we also suggest that future studies examining learning processes such as word learning should create scenarios in which children are tested at multiple time points, because this allows for a more precise focus on the specific learning processes, especially in shy children. On the other hand, given the high incidence of shyness as a normal variation in human personality in the overall population (Zimbardo et al., 1975; Kagan, 1994; Schmidt et al., 2020), our results also demonstrate that a nuanced assessment of shyness (e.g., parental assessment via a standardized questionnaire) is preferable, especially when it is central to the research question. In this vein, it is worth noting that temperamental characteristics are rarely collected in studies, and tests assessing children’s linguistic and cognitive abilities are often administered by (almost) unfamiliar interaction partners.

We postulate that addressing children’s individual differences and taking into account the personality of the interacting child can further guide future digital technologies and facilitate their integration into the educational landscape. However, at this point, we would like to be clear in our objective: We also see it as an ethical challenge to clarify whether future technologies should make automatic inferences about a child’s temperament. Instead, it is important to gain further insights into how children’s interactions with digital technologies such as social robots depend on their individual differences in order to enable educators and teachers to design future learning scenarios that allow all children to participate.

In future work, it would also be of interest to explore the effects of other dimensions of temperamental traits on interaction behavior such as susceptibility to frustration or attention span/task persistence. This would shed further light on how individual adaptation to the learner and appropriate learning environments can be designed in the digital world of the future.

## Data Availability

Based on the options within consent to future reuse of their data by other researcher that was given by the caregivers, the datasets presented in this article are not readily available and will not be made publicly available. Requests to access the datasets should be directed to Nils F. Tolksdorf, nils.tolksdorf@upb.de.
